# Impact of Enzymatic Hydrolysis and Heat Inactivation on the Physicochemical Properties of Milk Protein Hydrolysates

**DOI:** 10.3390/foods11040516

**Published:** 2022-02-11

**Authors:** Alice Gruppi, Maria Dermiki, Giorgia Spigno, Richard J. FitzGerald

**Affiliations:** 1 Department for Sustainable Food Process (DiSTAS), Università Cattolica del Sacro Cuore, Via Emilia Parmense 84, 29122 Piacenza, Italy; alice.gruppi@unicatt.it; 2 Department of Biological Sciences, School of Natural Sciences, University of Limerick, V94 T9PX Limerick, Ireland; Dermiki.Maria@itsligo.ie (M.D.); dick.fitzgerald@ul.ie (R.J.F.); 3 Faculty of Science, Institute of Technology Sligo, F91 YW50 Sligo, Ireland

**Keywords:** degree of hydrolysis, milk protein concentrate, molecular mass distribution, sodium caseinate, turbidity, viscosity, whey protein

## Abstract

This study determined the physicochemical properties (apparent viscosity (η_app_), turbidity (A_550nm_), particle size and molecular mass distribution) of hydrolysates generated from whey protein concentrate (WPC), milk protein concentrate (MPC) and sodium caseinate (NaCN), following incubation with Debitrase HYW20™ and Prolyve™ at 50 °C, pH 7.0 for 1 and 4 h, before and after heat inactivation (80 °C for 10 min). The degree of hydrolysis (DH) increased with incubation time, giving values of 6.56%, 8.17% and 9.48%, following 1 h hydrolysis of WPC, MPC and NaCN with Debitrase HYW20™, and 12.04%, 15.74% and 17.78%, respectively, following 4 h incubation. These DHs were significantly higher compared to those obtained following 4 h incubation with Prolyve™. Hydrolysis with Debitrase HYW20™ gave >40% of peptides with molecular masses < 1 kDa for all substrates, which was higher than the value obtained following hydrolysis with Prolyve™. The effect of hydrolysis on the physicochemical properties was substrate dependent, since η_app_ decreased in WPC and NaCN hydrolysates, particle size decreased for all the substrates, with aggregate formation for MPC, and turbidity decreased in WPC and MPC hydrolysates, while it increased in NaCN hydrolysates. The physical properties of the hydrolysates were influenced by the enzyme thermal inactivation step in a DH-dependent manner, with no significant effect on turbidity and viscosity for hydrolysates at higher DHs.

## 1. Introduction

Dairy proteins are commonly used as ingredients in complex food systems, either for structuring or for nutritional purposes [1]. The enzymatic hydrolysis of dairy proteins can enhance their techno- and bio-functional properties [2,3,4,5]. Whey protein concentrate (WPC), milk protein concentrate (MPC) and sodium caseinate (NaCN) represent some of the most used, and therefore, the most extensively studied dairy protein ingredients. Specifically, NaCN is utilised due to its foaming and emulsifying properties [6], while whey protein ingredients are utilised for their gelation and emulsification properties, and their good solubility at acid pH [7,8]. The application of MPCs [9], with high protein contents (>70%), may be impacted by the variability in their solubility properties [10,11,12].

Milk protein hydrolysates exhibit improved techno-functional properties compared to the intact proteins, thereby enhancing their applications as ingredients in different food preparations. Several studies have demonstrated an increase in protein solubility following enzymatic hydrolysis [13,14,15,16,17], as reported by Ryan et al. [18] who found that the hydrolysis of milk protein isolate, with Flavourzyme, Neutrase and Protamex, led to increased solubility at pH 4.0–7.0. Moreover, hydrolysis can lead to significant improvements in the foaming, gelling and emulsifying properties of whey protein hydrolysates [19,20,21,22] of casein hydrolysates [23] and milk protein hydrolysates [24,25] in comparison with the intact proteins. This improvement in functional properties may be attributed to changes in the secondary structure and to a decrease in molecular mass following hydrolysis [26]. In many cases, enzymatic hydrolysis of milk proteins can lead to improved bio-functional properties, due to the formation of peptides with, e.g., angiotensin converting enzyme-inhibitory activity, antidiabetic or antimicrobial activities [2], which are not biologically active when they are in the parent protein [2]. Whey proteins can also give rise to bioactive peptides once the primary structure is hydrolysed; these peptides can display different bioactivities, such as antioxidative activity, be more effective in treating tumours in some cancers and can inhibit ACE activity in vitro [27]. 

The properties of milk protein hydrolysates depend on the conditions under which they have been generated [3,4], such as the enzymes used, since different enzymes, depending on their specificity, will result in the formation of peptides with varying molecular masses and hydrophobicity [3]. Furthermore, hydrolysate properties are dependent on the pH employed during hydrolysis [28], hydrolysis time, incubation temperature [29], enzyme to substrate ratio [30] and total solids [31]. During their manufacture, hydrolysates are usually subjected to an enzyme heat inactivation step, prior to concentration and spray-drying. Therefore, when generating hydrolysates, it is important to consider the impact of both the hydrolysis and heat inactivation conditions on the properties of the hydrolysates generated, prior to and following drying. This is due to the fact that changes in the viscosity, turbidity and the formation of aggregates, during hydrolysis and subsequent enzyme thermal inactivation, can affect the heat and mass transfer properties during various processing steps. Therefore, it is expected that hydrolysis parameters, such as the enzyme employed, the duration of hydrolysis along with heat inactivation, will impact the properties of the hydrolysates generated using different substrates. 

The aim of this study was to investigate the impact of the enzyme preparation and incubation time on the physicochemical properties (DH, η_app_, turbidity (A_550nm_), particle size and molecular mass distribution) of hydrolysates, generated from WPC, MPC and NaCN using Debitrase™ and Prolyve™. The impact of enzyme heat inactivation on hydrolysate particle size, η_app_ and turbidity was also investigated. 

## 2. Materials and Methods

### 2.1. Materials

The milk protein substrates NaCN (Arrabawn Co-Operative Creamery, Nenagh, Ireland), MPC (Kerry Ingredients, Listowel, Ireland) and WPC (Carbery, Ballineen, Ireland) had protein contents of 85%, 84% and 82% (*w*/*w*), respectively, and were kindly provided by the above manufacturers. The food-grade proteolytic preparation Debitrase HYW20™ was kindly provided by Rhodia Ltd. (Cheshire, UK), and Prolyve 1000™ was provided by Lyven Enzymes Industrielles (Caen, France). 2,4,6-Trinitrobenzenesulfonic acid (TNBS) was obtained from Pierce Biotechnology (Medical Supply, Dublin, Ireland) and all other chemicals and reagents were purchased from Sigma Chemical Company Ltd. (Dublin, Ireland).

### 2.2. Methods

#### 2.2.1. Generation of Enzymatic Hydrolysates

**Figure 1:**
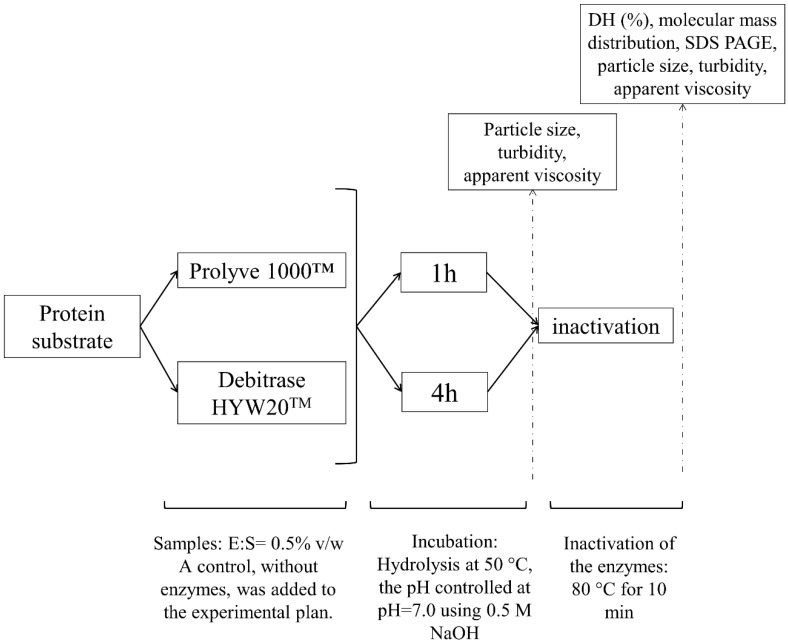
Schematic representation of the experimental approach. The initial substrate concentration for milk protein concentrate (MPC) and sodium caseinate (NaCN) was 8% (*w*/*w*) total solids and for whey protein concentrate (WPC) it was 10% (*w*/*w*) total solids in distilled water. Note: DH: degree of hydrolysis; E:S: enzyme to substrate ratio (*v*/*w*), SDS PAGE: Sodium dodecyl sulphate polyacrylamide gel electrophoresis.

The hydrolysis procedure was based on the protocol employed by Dermiki and FitzGerald [29] with some adaptations. Substrate suspensions with different concentrations were prepared based on their solubility. MPC and NaCN were dissolved at 8% (*w*/*w*) while WPC was reconstituted at 10% (*w*/*w*) in distilled water at room temperature and then gently stirred at 5 °C for 16 h to aid hydration. Before hydrolysis, the temperature of the substrate samples was adjusted to 50 °C and maintained at this temperature for 1 h prior to initiating the hydrolysis reaction. The pH of the substrate solutions was adjusted to pH 7.0 using 0.5 M NaOH. The enzyme (Debitrase HYW20™ or Prolyve 1000™) was added at an enzyme to substrate ratio (E:S) of 0.5% (*v*/*w*) while hydrolysis was carried out at 50 °C, using a magnetic stirrer (Fisherbrand, Fisher Scientific, Dublin, Ireland) set at 500 rpm and the pH of the hydrolysis reaction was controlled at pH 7.0 using a pH-stat (Titrando 843, Tiamo 1.4 Metrohm, Dublin, Ireland) by the addition of 0.5 M NaOH. Control samples of MPC, WPC and NaCN, without enzymes, were included in the experimental plan. The volume of all samples (hydrolysates and unhydrolyzed protein samples without the addition of enzyme) was adjusted with distilled water to achieve the same final total solids concentration (7.99% (*w*/*w*) for NaCN and MPC; and 9.99% for WPC). Enzyme inactivation was conducted by heating the solutions at 80 °C for 10 min in a water bath as reported by Dermiki and FitzGerald [29]. Hydrolysates were then frozen and stored at −20 °C until further analysis, unless stated otherwise. Figure 1 provides a schematic overview of the experimental approach employed.

Particle size distribution, turbidity and η_app_ analyses were determined before and after heat inactivation on the day of hydrolysate production while DH and molecular mass distribution analyses were performed on the heat-treated hydrolysates (Figure 1). 

#### 2.2.2. Determination of DH

The spectrophotometric TNBS assay was used to determine the DH (%) as reported by Adler-Nissen [32] with the modifications of Le Maux et al. [28]. Samples (hydrolysed and unhydrolyzed control samples after heat inactivation) were diluted in 1% (*w*/*v*) SDS to a final protein concentration/protein equivalent of 5% (*w*/*v*) and prior to incubation at 50 °C for 30 min. Then,10 μL of the test hydrolysate samples and leucine standards (0, 2, 5, 7, 14, 21, 28 and 56 mg of nitrogen/L) were loaded onto a 96-well plate with 160 μL TNBS working solution (0.05% TNBS (*w*/*v*) and a 1:1 mixture of preheated (50 °C) water and 0.2125 M phosphate buffer pH 8.2). The plate was incubated at 50 °C for 1 h in a Synergy™ HT plate reader (BioTek Instruments Limited, Bedfordshire, UK) and the absorbance at 420 nm was recorded. The DH was calculated using Equation (1), as follows:

(1) $$\mathrm{DH}\;\left(\%\right)=\frac{{\mathrm{AN}}_{\mathrm{sample}}-{\mathrm{AN}}_{\mathrm{unhydrolysed}\;\mathrm{sample}}}{\mathrm{Npb}}$$

where AN_sample_ is the amino nitrogen content of the protein hydrolysate (mg/g protein), AN_unhydrolyzed sample_ is the amino nitrogen content of the protein substrate before hydrolysis (mg/g protein) and Npb is the nitrogen content of the peptide bonds in the protein substrate (mg/g protein); values of 100, 123.3 and 112.1, respectively, were used for MPC [24], WPC [7] and NaCN [33]. Analysis was conducted in triplicate (n = 3).

#### 2.2.3. Particle Size Distribution

Particle size distribution of the hydrolysates and controls was determined by laser light scattering as described by Le Maux et al. [34].

A Mastersizer 2000 with a Hydro 2000S dispersion system (Malvern Instruments, Worcestershire, UK) was used to analyse the particle sizes of the samples. Laser obscuration between 5–10% was obtained using the dispersion unit before each measurement. The particle and the dispersant refractive index used were 1.52 and 1.33, respectively. Each sample was measured in triplicate. Analysis of the results was performed using the general-purpose model available from the Malvern software. The particle size distributions were expressed as the cumulative weight (%) per volume moment mean diameter of the particles (D_3,2_ µm). 

#### 2.2.4. Molecular mass Distribution

Gel permeation high-performance liquid chromatography (GP-HPLC) was used to determine the molecular mass distribution of the samples at a concentration of 0.25% (*w*/*v*) protein/protein equivalent as described by Nongonierma and FitzGerald [35]. Samples were filtered through 0.2 μm PTFE filters prior to injection. Aliquots (20 µL) of diluted sample were injected onto a TSK G2000 SW separating column (600 × 7.5 mm ID) (Tosoh 157 Bioscience, Stuttgart, Germany). Separation took place by isocratic elution using 0.1% (*v*/*v*) trifluoroacetic acid (TFA), 30% (*v*/*v*) acetonitrile in H_2_O, at a flow rate of 1 mL/min. Detector response was monitored at 214 nm [35]. A calibration curve was generated using the average retention times of the standards (BSA (67,500 Da), β-lactoglobulin (36,000 Da), α-lactalbumin (14,200 Da), aprotinin (6500 Da), bacitracin (1400 Da), Leu-Trp-Met-Arg-OH (605 Da), Asp-Glu (262 Da), Tyr (181 Da)). Molecular mass distributions were obtained by integrating the area under the curve corresponding to the average retention time of the different molecular masses at 10, 5 and 1 kDa.

#### 2.2.5. Turbidity (A_550nm_) Measurements

Turbidity was evaluated as described by O’Loughlin et al. [36]. Samples were diluted to 0.1% (*w*/*v*) protein/protein equivalent with distilled water and vortexed to prevent immediate separation, the absorbance (200 μL sample volume) was then read at 550 nm at room temperature using a Synergy™ HT plate reader. All samples were analysed in triplicate (n = 3).

#### 2.2.6. Determination of Apparent Viscosity (η_app_) 

The samples were equilibrated at 50 °C and the η_app_ was measured using a Brookfield DVII+ LV (Brookfield Engineering Laboratories, Middleboro, MA, USA) viscometer, fitted with an ultra-low adaptor (ULA). Measurements were conducted at a defined shear rate, i.e., 112 s^−1^ (or rotational speed of the spindle at 100 rpm). The ULA adaptor was connected to a Brookfield refrigerated circulating water bath (model TC-500) by an ULA-40Y water jacket in order to control the temperature at 50 °C during measurements as described by Dermiki and FitzGerald [29].

#### 2.2.7. Statistical Analysis

All statistical analyses were conducted using XLStat statistical software (XLStat, 2020.1.3.65324, Addinsoft, New York, NY, USA) [37]. Values presented are the mean of three replicates ± standard deviation, unless otherwise stated. The standardised residuals were calculated and were normally distributed. Normality was tested using the Shapiro–Wilk test which is best suited for small sample sizes. Data were also tested for homogeneity by plotting scatterplots of the residuals against predictors. These tests were all conducted as part of the ANOVA analysis using XLStat [37]. One-way analysis of variance (ANOVA), two-way ANOVA or full factorial design was used to test the effect of one, two or more factors (enzyme, incubation time, heated or unheated) on the responses studied, respectively. When significance was noted, comparison of means was conducted by employing a Tukey post-hoc test. Significance was determined at *p* < 0.05.

## 3. Results and Discussion

### 3.1. Hydrolysis of Milk Protein Substrates

The following two commercially available proteolytic preparations were used in the current study: Debitrase HYW20™ and Prolyve 1000™. Debitrase HYW20™ is an enzyme preparation derived from *Aspergillus oryzae* and *Bacillus spp.* (rich in exopeptidase and with proteases), which has been shown to produce hydrolysates with reduced bitterness [38,39], while Prolyve 1000™ is a *Bacillus licheniformis* proteinase, which does not cause gelation [31] and has also been reported to show decreased bitterness compared to Alcalase™, another commonly used *Bacillus licheniformis* proteinase [35].

According to Cui et al. [17], the treatment with Protamex, which has both endo- and exo-protease activity, led to the hydrolysates with the lowest levels of bitterness, compared with Alcalase (which preferentially hydrolyses peptide bonds containing aromatic amino acid residues), and Flavouryme (which is produced from *Aspergillus oryzae,* as for Debitrase HYW20™). However, MPC treated with Alcalase presented higher DH than those obtained by Protamex and Flavourzyme.

Table 1 shows the DHs obtained for the three substrates (WPC, MPC and NaCN) after incubation for 1 and 4 h with Debitrase and Prolyve. A two-way ANOVA was conducted to investigate the effect of enzyme and incubation time on the DH for each substrate. As expected from previous studies [40], there was an effect of incubation time on DH, with higher DHs being observed after 4 h incubation for all substrates with both enzymes. When considering the effect of the enzyme, no effect was observed after 1 h incubation with a DH of approximately 7% being reached with both enzymes for WPC and MPC, whereas in the case of NaCN, a higher DH % (9.48 ± 0.88) was observed on incubation with Debitrase. When comparing the three substrates, a higher DH was observed for NaCN and MPC after 4 h incubation (17.78 ± 1.00 and 15.74 ± 1.36, respectively, with Debitrase), possibly due to the fact that caseins are more susceptible to hydrolysis compared to whey proteins [22] and these two substrates predominantly contain casein. The higher susceptibility of casein to hydrolysis could be attributed to its open or disordered structure, while whey proteins have a globular structure in their native state [41].

Prolyve is a *Bacillus licheniformis* proteolytic preparation with a broad substrate specificity, which may explain the relatively high DH (around 7%) obtained with all three substrates, even after 1 h of incubation. This enzyme preparation preferentially cleaves at the carboxyl side of hydrophobic amino acid residues [42]. Debitrase HYW20 contains *Aspergillus oryzae* exopeptidase activity, allowing it to generate hydrolysates rich in free amino acids. Moreover, due to the presence of proteases from *Bacillus* spp., Debitrase has broad specificity [43], resulting in relatively high DH values. When comparing the DH of the hydrolysates generated after 4 h of incubation, Debitrase resulted in higher DH values compared to Prolyve with all substrates. These findings are in agreement with those of Spellman et al. [7], who reported higher DH values for whey protein hydrolysates generated using Debitrase, compared to those generated using Alcalase, which is also a *Bacillus licheniformis* proteinase like Prolyve. In other studies [44], the hydrolysis of NaCN with Debitrase resulted in a relatively low DH compared to a range of other enzymes; however, the DH was calculated from the volume of NaOH consumed during hydrolysis, whereas in the current study, it was determined using the TNBS method. Moreover, different reaction times and pH values were employed in the two studies. There appears to be no information on the hydrolysis of MPC using Debitrase or Prolyve. As previously mentioned, Cui et al. [17] hydrolysed MPC using Alcalase, Protamex and Flavourzyme, and obtained DH around 15.3% with Alcalase after 2 h of hydrolysis. This value is significantly higher than the DH obtained in the current study using Prolyve, an enzyme with similar activity to Alcalase. These differences could be due to the different ways of measuring the DH (TNBS vs. o-phthaldialdehyde (OPA)) or possibly different hydrolysis conditions of pH and temperature.

**Figure 2:**
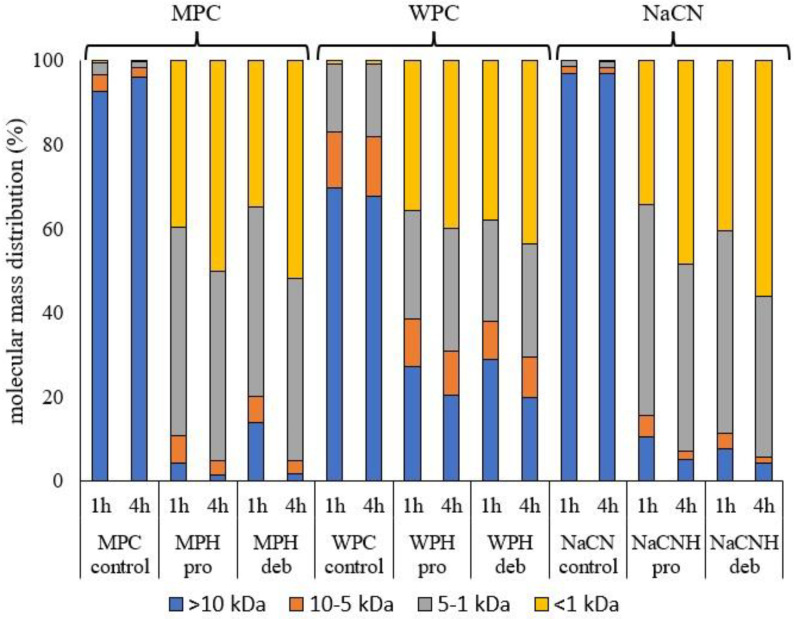
Molecular mass distribution profiles of the unhydrolyzed whey protein concentrate (WPC), sodium caseinate (NaCN) and milk protein concentrate (MPC) control samples and their corresponding hydrolysates (respectively, WPH, NaCNH and MPH) generated using Prolyve 1000™ (WPH pro, NaCNH pro, MPH pro) and Debitrase HYW20™ (WPH deb, NaCNH deb, MPH deb) following 1 and 4 h incubation at 50 °C. kDa: kilo Dalton.

Protein hydrolysis was further confirmed using GP-HPLC, as seen in Figure 2, showing the breakdown of high molecular mass (>10 kDa) components representing the intact protein during the hydrolysis process. The molecular mass distribution of the peptides generated during hydrolysis may impact the nutritional properties of the hydrolysates, e.g., their bioavailability and digestibility [45], along with techno-functional properties, such as solubility, emulsification, foaming and gelation. This has been related to lower molecular weight components exhibiting better interfacial diffusivity compared to large biopolymers [46], even though higher DH values may result in the loss of emulsifying properties [47,48]. 

As seen in Figure 2, enzymatic treatment clearly results in a decrease in molecular mass, with higher percentages of lower molecular mass components in the hydrolysates compared to the corresponding controls. As expected from the results of the DH analyses presented in Table 1, all the substrates show a higher proportion of lower molecular mass components after 4 h incubation. As already mentioned, Debitrase contains exopeptidase activity, and its hydrolysates are expected to contain higher concentrations of short peptides/free amino acids. On the other hand, hydrolysis by *B*. *licheniformis* proteases, such as Prolyve, is expected to release peptides without free amino acids [49]. Debitrase hydrolysates had a higher proportion of components with lower molecular mass (<1 kDa) compared to Prolyve hydrolysates, e.g., in the case of NaCN (56% versus 49%) and WPC (43.5% versus 40%). However, in the case of the MPC hydrolysates, both enzymes produced a similar amount (~50%) of components, with a molecular mass < 1 kDa. The observation that the percentage of molecules with low molecular mass is lower in WPC hydrolysates is in agreement with the differences observed in DH (see Table 1) and the fact that casein is more susceptible to hydrolysis compared to whey proteins. Moreover, the relatively high percentage of low molecular weight compounds in the NaCN and MPC hydrolysates is in agreement with the findings of McDonagh and FitzGerald [43], who reported high percentages of low molecular weight components (<3 kDa) when Debitrase was used. 

### 3.2. Physicochemical Characteristics of the Hydrolysates 

#### 3.2.1. Particle Size

**Figure 3:**
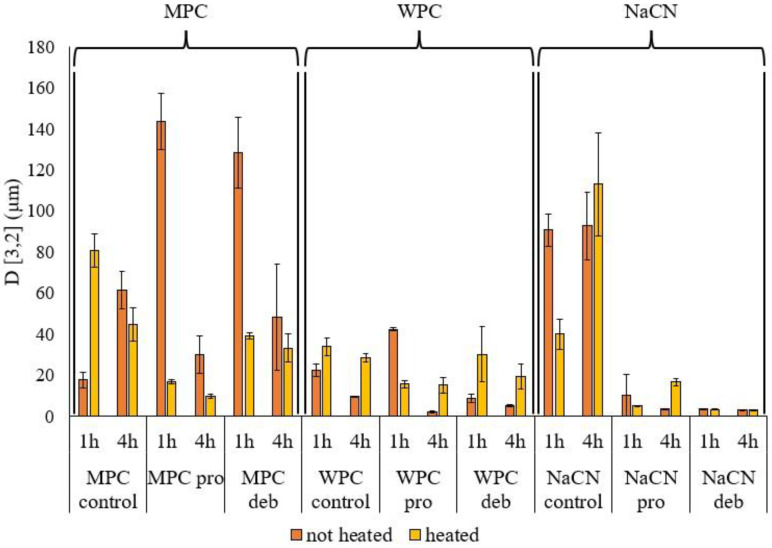
Particle size distribution expressed as volume moment mean diameter D[3,2] for the unhydrolyzed (control) protein substrates, milk protein concentrate (MPC), whey protein concentrate (WPC) and sodium caseinate (NaCN) and their corresponding hydrolysates generated using Prolyve 1000™ (pro) and Debitrase HYW20™ (deb) following 1 and 4 h of incubation at 50 °C before (not heated) and after heat inactivation at 80 °C for 10 min (heated).

**Figure A1:**
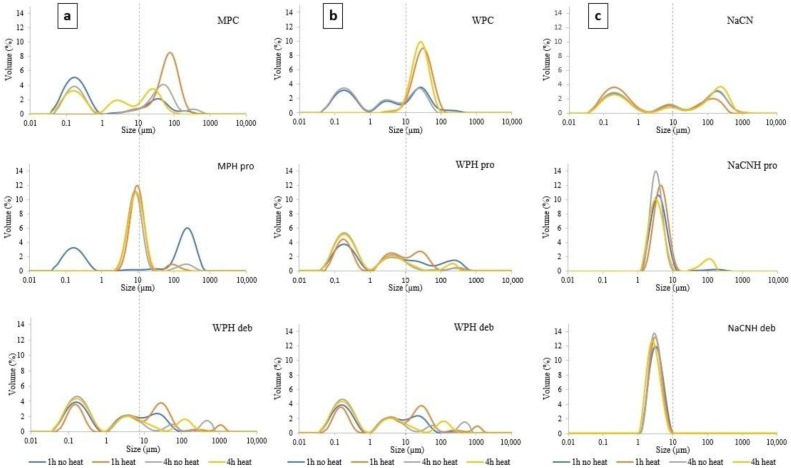
Particle size distribution profiles of intact protein substrates and their corresponding hydrolysates (H). ((**a**): milk protein concentrate (MPC); (**b**): whey protein concentrate (WPC); (**c**): sodium caseinate (NaCN)) generated using Prolyve 1000™ (pro) (MPH pro, WPH pro, NaCNH pro) and Debitrase HYW20™ (deb) (MPH Deb, WPH deb, NaCNH deb) after 1 and 4 h of incubation at 50 °C before (no heat) and after heat inactivation at 80 °C for 10 min (heat).

Figure 3 represents the volume moment mean diameter D[3,2] of particles in suspension for WPC, MPC and NaCN, before and after hydrolysis, and after heat inactivation showing an influence of both substrate and enzyme type. For MPC, the particle size decreased with incubation time for both the hydrolysates and the intact protein. The latter may be related to the increased solubility of MPC with increasing incubation time. Moreover, at 1 h incubation, the particle size of the MPC hydrolysates was higher compared to the intact protein, possibly due to aggregation between the peptides in solution, while subsequent heating resulted in a decrease in D[3,2]. This was more evident at low DH and short incubation times for both enzymes. Cui et al. [17] reported increased particle size of hydrolysates of MPC, generated using Alcalase, Protamex and Flavourzyme, compared to the intact protein. In the study by Cui et al. [17] changes in particle size during incubation were enzyme-dependent. They had not reported, however, the effect of heat inactivation on the particle size of MPC hydrolysates. In the case of NaCN, the particle size decreased on hydrolysis, regardless of the enzyme used, even after 1 h incubation. For the WPC hydrolysate, particle size decreased with incubation time for both enzymes but with no clear trend, as seen in Figure 3. This may be attributed to the observation that in most cases, the particle size distribution was neither normal nor bimodal, especially for the MPC and WPC hydrolysates, for which Figure A1 shows how the particle size distributions changed during hydrolysis. For WPC, it was evident that the particle size decreased without aggregation during hydrolysis, with no differences due to treatment time and enzyme type. However, the heat inactivation step modified the particle size distributions, as seen in Figure 3. In the case of NaCN, no aggregate formation was observed for the hydrolysates generated using Prolyve, especially after 4 h of incubation, while subsequent heating resulted in the formation of aggregates. However, in the case of NaCN treated with Debitrase HYW20™, the particle size distributions were not affected by heating, as seen in Figure 3. The formation of aggregates during hydrolysis can impact the turbidity and the behaviour of hydrolysates as ingredients in complex food matrices [50].

#### 3.2.2. Turbidity

**Figure 4:**
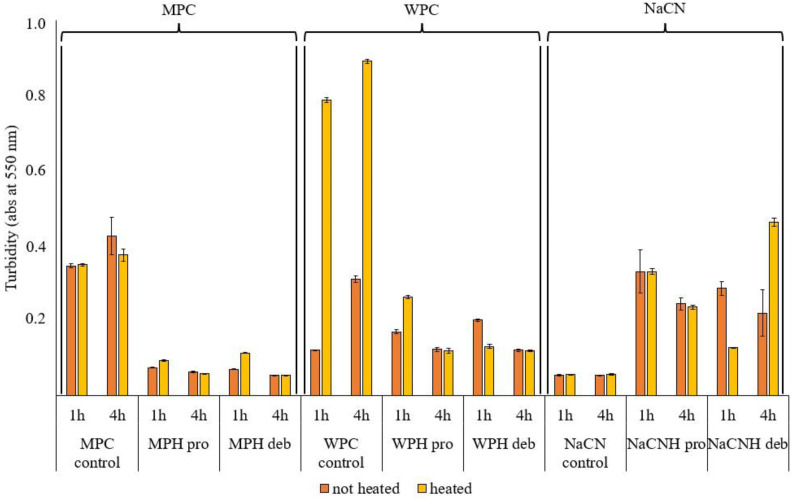
Turbidity expressed as absorbance at 550 nm for unhydrolyzed milk protein concentrate (MPC control), whey protein concentrate (WPC control) and sodium caseinate (NaCN control) control samples and their corresponding hydrolysates (H) incubated with Prolyve 1000™ (Pro) (MPH pro, WPH pro, NACNH pro) and Debitrase HYW20™ (Deb) (MPH deb, WPH deb, NaCNH deb) after 1 and 4 h of incubation at 50 °C before (not heated) and after heat inactivation at 80 °C for 10 min (heated).

**Figure A2:**
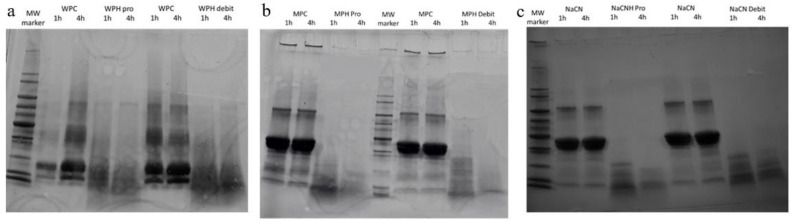
Sodium Dodecyl Sulphate Polyacrylamide Gel Electrophoresis (SDS page) of the intact protein and the corresponding hydrolysates. ((**a**): WPC; (**b**): MPC; (**c**): NaCN) generated using Prolyve (Pro) and Debitrase (Debit) after 1 h and 4 h of incubation.

The turbidity of protein solutions depends on protein concentration, the presence of non-dissolved particles, the particle size and particle number per volume unit [51]. Turbidity analysis of the samples, expressed as absorbance at 550 nm (Figure 4), indicated that the presence of aggregates (as confirmed by the particle size as measured using light scattering, seen in Figure 3 and Figure A1) increased the turbidity of NaCN after hydrolysis, as reported by Ewert et al. [52], while for WPC and MPC, the turbidity decreased after hydrolysis. The heat inactivation treatment significantly increased the turbidity of unhydrolyzed whey protein concentrate and the whey protein hydrolysate (WPH) generated using Debitrase after 1 h of hydrolysis. This could be attributed to the fact that Debitrase is an exopeptidase containing preparation, which at low DH, resulted in the formation of low molecular mass compounds, though there was still a significant amount of intact protein present (~30%), as shown in Figure 2. This result was further confirmed on SDS PAGE analysis (Figure A2), where a band of intact β-lactoglobulin was evident. Previous research has shown that the presence of β-lactoglobulin (β-lg) could lead to the formation of heat-induced aggregates, which in turn contribute to increased turbidity [31].

In the case of the WPH generated with Prolyve, heating had no impact on the turbidity in the 4 h hydrolysates, while at low DH (after 1 h of incubation), there was a decrease in turbidity with heating. This is an indication that there were no aggregates in the hydrolysate after heating, which corroborates further the findings from the particle size distribution analysis (Figure 3). The absence of aggregates, herein, is in agreement with Spellman et al. [38], who showed no aggregation in the WPHs generated using Prolyve in contrast to those generated with Alcalase, which is a *Bacillus licheniformis* enzyme preparation that has been reported to lead to WPC hydrolysate aggregation. Heating only affected the turbidity of unhydrolyzed WPC, as heating of whey at 80 °C can lead to aggregation, due to the presence of β-lg, which is subject to thermal denaturation and, consequently, the formation of aggregates [53]. SDS PAGE analysis (Figure A2) showed that β-lg was hydrolysed extensively in the case of the WPH generated using Prolyve, and this could be the reason why heat inactivation did not increase the turbidity of these hydrolysates.

In the case of MPC, turbidity was lower for the hydrolysates compared to intact MPC. After 4 h of hydrolysis, where highest DH was observed, the turbidity was lower, regardless of the enzyme used, possibly due to the low percentages of high molecular weight components, an indication that the intact protein had been hydrolysed (Figure 2). At low DH (after 1 h of incubation), the turbidity was affected by the inactivation treatment for both enzymes. Interestingly, there was a small increase in turbidity for the Prolyve MPC hydrolysates and a larger increase for the Debitrase MPC hydrolysates, which may be explained by the higher percentage of high MW components in Debitrase vs. Prolyve hydrolysates after 1 h incubation, as seen in Figure 2 (13.8% vs. 4.2%, respectively).

In the case of NaCN hydrolysates, turbidity increased for the hydrolysates compared to the unhydrolyzed control samples. This was also evident from the particle size distribution profiles (Figure A1), showing that the particle sizes increased for the hydrolysates generated using Prolyve, which could be due to the formation of aggregates. However, the significant increase in turbidity of the heated 4 h hydrolysates generated using Debitrase cannot be explained, taking into consideration the particle size distribution and the particle size of these samples, as seen in Figure A1 and Figure 3, respectively. This increase in turbidity could, however, be due to changes in solubility.

Changes in turbidity and particle size are indicative of the presence of aggregates or a decrease in solubility, which could be the case for the sodium caseinate hydrolysates (NaCNH) herein. Previous research has shown a decreased nitrogen solubility index at pH 7.0 for NaCN hydolysates generated using Protamex, a *Bacillus* proteinase, at low DH [14]. Low solubility at pH 6.0 and 7.0 (in the current study, all analyses were conducted at pH 7) was observed for NaCNHs generated with different enzymes [26,54]. As described by Flanagan and FitzGerald [14], this low solubility of the hydrolysates, compared to the intact protein, could be attributed to the formation of peptides with different pIs. High turbidity, due to low solubility, can affect the further processing of hydrolysates, which need, e.g., to be pumped and spray dried. Moreover, this can affect the food products that contain them, such as in the case of juices, or beverages in general, where turbidity can impact consumer acceptability [55]. Moreover, in the case of yoghurts, the presence of large protein aggregates could result in the formation of products with low storage moduli, yield stress, firmness and thickness [56], or could lead to the gelation of acid milk gels, as described by Gélebart et al. [57].

#### 3.2.3. Apparent Viscosity

**Figure 5:**
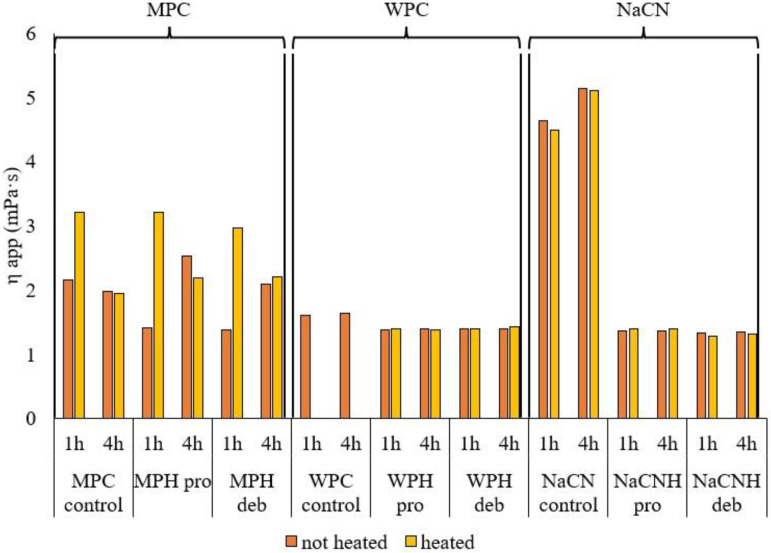
Apparent viscosity (η app) in mPa·s, measured at a shear rate of 122 s^−1^, of unhydrolyzed milk protein concentrate (MPC control), whey protein concentrate (WPC control) and sodium caseinate (NaCN control) control samples and their corresponding hydrolysates (H) before (no heated) and after heating at 80 °C for 10 min (heated). Hydrolysates were generated using Prolyve 1000™ (pro) and Debitrase HYW20™ (deb) after 1 and 4 h of incubation at 50 °C. Note: WPC unhydrolyzed control gelled on heating; therefore, the η_app_ could not be measured.

Figure 5 represents the findings on the η_app_ for the different samples. A decrease in viscosity after enzymatic hydrolysis has been widely demonstrated for most substrates in previous research [58,59]. The hypothesis that hydrolysis leads to a decrease in η_app_ has been confirmed for WPC and NaCN (Figure 5). A higher decrease in η_app_, compared to in MPC and WPC hydrolysates, was observed for the NaCN hydrolysates, for both enzymes used, while there were no significant differences in η_app_ of the MPC hydrolysate compared to the original MPC. In the case of NaCN, previous research reported a decrease in the apparent viscosity of hydrolysates having DH values ~10%, compared to intact NaCN [14]. The viscosity of NaCN and its hydrolysates generated using a *Bacillus* proteinase differed significantly at pH values close to the isoelectric point (pH = 4.0), while in the current study, all samples were tested at pH 7.0. In the case of whey proteins, previous studies observed gel formation of whey after limited hydrolysis with a *Bacillus licheniformis* protease [60], on extensive hydrolysis [61] or on heat treatment of whey protein hydrolysates [31]. However, this was not the case in the current study, where Prolyve, a *Bacillus licheniformis* enzyme, which does not induce gelation, was used [35].

Heating also had no effect on the viscosity of the WPC and NaCN hydrolysates, regardless of enzyme and incubation time. This is an important result, considering that an enzyme inactivation step, typically by heating, is required before the hydrolysates can be further processed. While careful control of the viscosity is important for processing, it is also of relevance for the application of hydrolysate ingredients in a range of products. Maintaining low viscosity can enhance heat and mass transfer during subsequent hydrolysate processing steps, such as pumping, concentration and drying. In terms of final product characteristics, careful control of the viscosity may lead to the development of desirable mouthfeel and textural properties.

In relation to MPC at low DH, the η_app_ increased after heat inactivation, while there were no significant differences in η_app_ after heating of the 4 h-hydrolysates regardless of the enzyme used. This could partly be explained by the molecular mass distribution profile shown in Figure 2 where in the case of 1 h incubation there was a significant amount of intact protein remaining compared to that in the 4 h hydrolysates. These results are in line with the MPH turbidity findings, as seen in Figure 4.

Since no previous studies have been conducted on the hydrolysis of MPC with Debitrase or Prolyve, the findings of the current study may only be discussed in relation to a study testing the hydrolysis of MPI using different enzymes [24]. Ryan et al. [24], who studied the changes in η_app_ for hydrolysates of milk protein isolate, at three different temperatures (25, 45 and 90 °C) and three different pH values (6.2, 6.8 and 7.2). These authors reported an effect of temperature and pH on η_app_. Moreover, the changes observed depended on the DH of the hydrolysates, which ranged from 15 to 37%, while the highest DH achieved in the current study with MPC was 15% (Table 1). It is difficult to compare the apparent viscosities of the current study with the findings of Ryan et al. [24], because of the different enzymes used and the different pHs at which the viscosity was measured. However, at lower DH, in the present study, we observed a decrease in η_app_ of MPH compared to MPC. Heating at 80 °C for 10 min resulted in an increase in η_app_, possibly due to a decreased heat stability (as reported by Ryan et al. [24], between pH 6.2 and 7.4), possibly due to changes in casein micelle structure. These changes may be linked to the observed changes in turbidity. Further hydrolysis (4 h) increased the viscosity of the unheated MPC hydrolysates. However, heating did not alter the viscosity of these samples. The η_app_ in this case for the heat-treated samples was measured after the samples were heated at 80 °C and were then cooled to 50 °C, to conduct all measurements at the same temperature. This cooling step may impact the η_app_ values, as shown by Ryan et al. [24], who reported a viscosity increase upon the cooling of milk protein isolate (MPI) control samples and low DH MPI hydrolysates, generated using a variety of enzymes (i.e., by ~15% with Neutrase and Flavourzyme and by ~17% with Protamex).

## 4. Conclusions

The current study explored the impact of hydrolysis conditions, such as enzyme preparation, incubation time and heat inactivation, on the physicochemical properties of hydrolysates, generated from different milk protein substrates (WPC, MPC and NaCN). The two enzymes used, Debitrase and Prolyve, were chosen on the basis that they may lead to the generation of hydrolysates with reduced bitterness. Following 1 h incubation, the degree of hydrolysis was higher for NaCN compared to the other substrates and, while there was no enzyme effect on the DHs of WPC and MPC hydrolysates, hydrolysis with Debitrase resulted in a higher DH for NaCN. The effect of hydrolysis on the physicochemical properties depended on the substrate. For example, η_app_ decreased in WPC and NaCN hydrolysates, particle size decreased for all the substrates, with aggregate formation for MPC, and turbidity decreased in WPC and MPC hydrolysates but increased in NaCN hydrolysates. Viscosity, turbidity and particle size changes were substrate- and incubation time-dependent and, to a lesser extent, also enzyme-dependent. Heat inactivation, an essential step during the processing of hydrolysates, impacted the hydrolysate physicochemical properties in different ways, depending on both the DH and the starting substrate. At longer incubation times, which also resulted in higher DHs, heat inactivation of the hydrolysates did not significantly impact the turbidity and viscosity of the hydrolysates, which is of significant industrial importance, when considering the further processing of hydrolysates. The findings reported herein, may help in the design of enzymatic processing approaches for the generation of hydrolysate ingredients from different milk protein starting substrates.

## Figures and Tables

**Table 1:** Degree of hydrolysis (DH, %) of milk protein concentrate (MPC), whey protein concentrate (WPC) and sodium caseinate (NaCN) hydrolysates as a function of enzyme preparation, Prolyve 1000™ (Pro) or Debitrase HYW20™ (Deb), and incubation time. Values presented are mean ± standard deviation (n = 3).

Substrate	Enzyme	DH (%) at Different Incubation Times	
		1 h	4 h
MPC	Control	0.92 ± 0.21 ^e^	1.04 ± 0.37 ^e^
	Pro	7.05 ± 0.40 ^c^	11.65 ± 0.98 ^b^
	Deb	8.17 ± 0.58 ^c^	15.74 ± 1.36 ^a^
WPC	Control	2.11 ± 0.15 ^d^	2.26 ± 0.07 ^d^
	Pro	7.12 ± 0.50 ^c^	9.54 ± 1.09 ^b^
	Deb	6.56 ± 0.38 ^c^	12.04 ± 0.73 ^a^
NaCN	Control	0.30 ± 0.26 ^e^	0.66 ± 0.26 ^e^
	Pro	7.18 ± 0.67 ^d^	11.67 ± 0.67 ^b^
	deb	9.48 ± 0.88 ^c^	17.78 ± 1.00 ^a^

Note: ^a, b, c, d, e^ superscripts with a different letter across each substrate represent means which are significantly different at a significance level *p* < 0.05 as determined using two-way ANOVA and post-hoc Tukey test.

## Data Availability

The data that support the findings of this study are available from the corresponding author upon reasonable request.

## Appendix A

SDS polyacrylamide gel electrophoresis (SDS PAGE): For determination of the electrophoretic profile, the samples (hydrolysates and unhydrolyzed control after heat inactivation) were resuspended in a solution of 1% (*w*/*v*) SDS, in order to dissociate the proteins. The sample buffer contained β-mercaptoethanol to carry out the electrophoresis under reducing conditions. The running buffer used was Tris-glycine-SDS (25 mM Tris, 192 mM glycine and 0.1% SDS, pH 8.6). Gels were run at a constant voltage of 150 mV for 60 min. Mini-PROTEAN TGX precast gels (4–20% resolving gel, Bio-Rad Laboratories Inc., Hercules, CA, USA) were used on a Mini Protean II system (Bio-Rad), according to the manufacturer’s instructions. The quantity of protein added to each well was 15 μg. The molecular masses of the proteins were estimated by reference to the relative migration of the molecular mass standards, which had a wide range of molecular weights (6500–200,000 Da, Sigma-Aldrich, St. Louis, MO, USA). The gels were stained with Coomassie Blue R-250.
